# Pedestrian Detection Algorithm for Intelligent Vehicles in Complex Scenarios

**DOI:** 10.3390/s20133646

**Published:** 2020-06-29

**Authors:** Jingwei Cao, Chuanxue Song, Silun Peng, Shixin Song, Xu Zhang, Yulong Shao, Feng Xiao

**Affiliations:** 1 State Key Laboratory of Automotive Simulation and Control, Jilin University, Changchun 130022, China; caojw18@mails.jlu.edu.cn (J.C.); scx@jlu.edu.cn (C.S.); pengsilun@jlu.edu.cn (S.P.); xuz19@mails.jlu.edu.cn (X.Z.); 2 Taizhou Automobile Power Transmission Research Institute, Jilin University, Taizhou 225322, China; 3 School of Mechanical and Aerospace Engineering, Jilin University, Changchun 130022, China; songshx202@126.com; 4 Zhengzhou Yutong Bus Co., Ltd., Zhengzhou 450016, China; SYL20081243@126.com

**Keywords:** driving assistance, intelligent vehicle, YOLOv3, convolutional neural network, pedestrian detection

## Abstract

Pedestrian detection is an important aspect of the development of intelligent vehicles. To address problems in which traditional pedestrian detection is susceptible to environmental factors and are unable to meet the requirements of accuracy in real time, this study proposes a pedestrian detection algorithm for intelligent vehicles in complex scenarios. YOLOv3 is one of the deep learning-based object detection algorithms with good performance at present. In this article, the basic principle of YOLOv3 is elaborated and analyzed firstly to determine its limitations in pedestrian detection. Then, on the basis of the original YOLOv3 network model, many improvements are made, including modifying grid cell size, adopting improved k-means clustering algorithm, improving multi-scale bounding box prediction based on receptive field, and using Soft-NMS algorithm. Finally, based on INRIA person and PASCAL VOC 2012 datasets, pedestrian detection experiments are conducted to test the performance of the algorithm in various complex scenarios. The experimental results show that the mean Average Precision (mAP) value reaches 90.42%, and the average processing time of each frame is 9.6 ms. Compared with other detection algorithms, the proposed algorithm exhibits accuracy and real-time performance together, good robustness and anti-interference ability in complex scenarios, strong generalization ability, high network stability, and detection accuracy and detection speed have been markedly improved. Such improvements are significant in protecting the road safety of pedestrians and reducing traffic accidents, and are conducive to ensuring the steady development of the technological level of intelligent vehicle driving assistance.

## 1. Introduction

Since the beginning of the 21st century, the growth of the automobile industry has gradually changed people’s daily travel patterns. Despite the convenience brought by automobile technology, humans also face increasingly serious traffic safety issues. Studies have shown that subjective behaviors such as inattention, unresponsiveness, and impoliteness of drivers toward pedestrians can easily cause unnecessary casualties in traffic accidents, thereby posing a huge threat to human life and property [1,2,3,4,5]. With the substantial improvement of modern control technology and automotive technology, smart cars can assist or even completely replace drivers to perform the main driving operation, thereby providing a solution to traffic safety problems [6,7]. Pedestrian detection is an important aspect of the development of intelligent vehicles, which directly affects the driver’s road condition judgment. Smart cars obtain the actual road information around the vehicle in real time through the vehicle-mounted camera, and then uses the pedestrian detection technology to effectively detect pedestrian objects that appear in front of the vehicle, so that timely feedback and warning can be provided to the driver, and the driver take the correct driving operation to avoid the pedestrians. This is helpful to ensure the road safety of people and greatly reduce the traffic accident rate [8,9,10,11]. Therefore, this subject deserves further in-depth study. 

Pedestrian detection refers to the automatic detection of the presence of walking people from a collected detection image or video sequence, and accurate positioning of the pedestrian area. However, as pedestrians are non-rigid objects, complex backgrounds, different postures, changing light, and varying degrees of occlusion in actual road scenarios pose challenges to the accurate detection of pedestrians [12,13,14,15]. With the rapid development of computer science and artificial intelligence technology, pedestrian detection, as an important branch of computer vision, has attracted considerable research attention. Pedestrian detection research methods are generally divided into two categories, namely, traditional and deep learning-based detection methods. Traditional pedestrian detection methods are mostly implemented step by step based on statistical learning. First, effective feature extraction is conducted in the candidate region of the detection image, and then input to the classifier for discrimination, and finally output the results combined with the detection model [16,17,18]. Dollar et al. [19] proposed a research method for multi-scale pedestrian detection using fast feature pyramids based on aggregated channel features (ACF). This method first calculated the features of detection image by channel, and then obtained the final feature vector by integral histogram, which had a good detection effect on most visible light images. Gaikwad et al. [20] proposed a pedestrian detection method based on edge features, which effectively reduced the computational complexity of the feature classifier. However, the detection effect was poor when the edge features of pedestrians in the detected image were not obvious or clear. Liu et al. [21] effectively combined the linear kernel function with the two heterogeneous features of oriented gradient histogram and local binary pattern. The multi-view-pose part ensemble detector enhanced the expression ability of pedestrian features, exhibiting robust properties. Baek et al. [22] used kernel support vector machine (SVM) as a feature classifier for pedestrian detection, and trained and optimized it by genetic algorithm to obtain higher detection accuracy. In a word, the traditional pedestrian detection method has a good detection effect under a simple background. However, when the actual road scenario becomes complex, the detection image is blurry, or the pedestrian is in motion, the detection accuracy of this method decreases and is easily affected by environmental factors [23,24,25].

In recent years, deep learning models represented by convolutional neural network (CNN) have been successfully applied to the field of computer vision. As deep learning has a significant advantage of self-learning pedestrian characteristics, pedestrian detection methods based on deep learning have developed rapidly. Chen et al. [26] extracted the gradient features of pedestrians in a detection image based on deep CNN, input them to the SVM classifier for detection, and then achieved highly satisfactory detection results. Li et al. [27] adopted a scale-adaptive Fast RCNN framework that can effectively integrate large and small subnets, and had good adaptability to pedestrian detection at different scales. Ouyang et al. [28] proposed a joint deep learning framework for pedestrian detection, focusing on deformation and occlusion processing, and realized automatic interaction between related components, thereby showing competitive advantages in detection accuracy. Hou et al. [29] proposed a multispectral pedestrian detection algorithm that combines a single-shot detector framework with multispectral pixel-level image fusion methods, and the detection performance was further improved. Chu et al. [30] proposed a Syncretic-NMS algorithm for instance segmentation in object detection. Based on the traditional NMS algorithm, the bounding box was merged with its strongly related neighboring boxes, and the experimental results showed that Syncretic-NMS algorithm can effectively improve the accuracy of instance segmentation and adapt to different application scenarios. Recently, pedestrian detection methods based on deep learning are mainly divided into two-stage detection and one-stage detection methods. RCNN, SPP, Faster RCNN, and Mask RCNN are typical two-stage detection networks that have high detection accuracy. However, due to the high complexity of algorithms, long calculation time, and poor real-time performance, these networks cannot be effectively applied to the actual road scenarios [31,32,33]. OverFeat, SSD, and YOLO are typical one-stage detection networks. Furthermore, although these methods have high detection speed, they sacrifice a certain degree of detection accuracy and cannot effectively solve the problem of large network model parameters [34,35,36]. In general, many research methods have achieved positive research results on pedestrian detection technology, but different algorithms have advantages and disadvantages, and the detection performance is uneven. At present, there is no deep learning-based pedestrian detection method that can exhibit accuracy and real-time performance together when applied to complex road scenarios. Different algorithms still have different degrees of limitations, which is not conducive to the further development of the technological level of intelligent vehicle driving assistance. Therefore, improving the pedestrian detection algorithm in view of the above problems is necessary.

In this study, a pedestrian detection algorithm for intelligent vehicles in complex scenarios is proposed. First, the basic principle of YOLOv3 is elaborated and analyzed to determine its limitations in pedestrian detection. Then, on the basis of the original YOLOv3 network model, many improvements are made, including modifying grid cell size, adopting improved k-means clustering algorithm, improving multi-scale bounding box prediction based on receptive field, and using Soft-NMS algorithm. Finally, based on INRIA person and PASCAL VOC 2012 datasets, pedestrian detection experiments are conducted to test the performance of the algorithm in various complex scenarios. By comparing the detection performance with other algorithms, the performance of the proposed algorithm is evaluated.

The rest of this paper is organized as follows: In Section 2, based on the basic principle of YOLOv3, the limitations of its application to pedestrian detection are determined. In Section 3, the original YOLOv3 network model is improved. In Section 4, pedestrian detection experiments are conducted based on relevant datasets, the detection effect is observed, and the performances of the algorithms are compared. Section 5 summarizes the conclusions and provides directions for future work.

## 2. YOLOv3 Network Model

### 2.1. Basic Principle of YOLOv3

YOLO, which stands for “you only look once,” is a classic one-stage object detection algorithm based on deep learning. YOLO was first proposed by Joseph Redmon at the International Conference on Computer Vision and Pattern Recognition in 2016 [37,38]. After continuous improvement and upgrading of the algorithm in recent years, YOLOv3 version has been released. This algorithm innovatively integrates candidate region selection and object recognition into a single deep neural network, transforming object detection into a regression problem so that the network can directly output the detection results. Therefore, the detection speed of this algorithm is significantly faster than that of the general two-stage object detection algorithm.

YOLOv3 draws on the network design ideas of GoogLeNet and ResNet. On the basis of YOLO and YOLOv2, the third version uses Darknet-53 as backbone network for feature extraction. According to feature pyramid network (FPN), YOLOv3 uses the feature map fusion method to achieve multi-scale prediction. Softmax classifier is no longer used to classify each box, but a logistic classifier is employed to complete the multi-label classification task. Darknet-53 is a deep network consisting of 53 layers of CNNs, and a large number of convolution kernels with sizes of 1 × 1 and 3 × 3 are used. After each CNN, a batch normalization operation and Leaky ReLU activation function are followed to prevent over-fitting. In addition, the network also adds a residual structure, which sets up a shortcut link between several layers so that it can increase the depth of the network without reducing its accuracy, and solves the problem of gradient explosion or gradient disappearance that can easily occur due to the excessive depth of the network. Table 1 lists the basic parameters of the Darknet-53 network framework.

YOLOv3 extends the anchor box idea of YOLOv2, and uses dimension clustering to predict the bounding box. The determination of a single bounding box requires four values, namely, coordinates $b_{x}$ and $b_{y}$, width $b_{w}$, and height $b_{h}$. If the coordinate of the upper left corner of the network element in the feature map is $\left(c_{x},c_{y}\right)$, and the width and height of the anchor box are $p_{w}$ and $p_{h}$, then the position and size of the predicted bounding box can be expressed as follows:

(1) $$\begin{array}{c} b_{x}=\sigma (t_{x})+c_{x}\\ b_{y}=\sigma (t_{y})+c_{y}\\ b_{w}=p_{w}e^{t_{w}}\\ b_{h}=p_{h}e^{t_{h}} \end{array}$$

where $t_{x}$ and $t_{y}$ are the coordinate offset values, $t_{w}$ and $t_{h}$ are the scaling, and σ is the confidence with a value range between 0 and 1.

To predict each detection image, YOLOv3 uses three scale feature maps with sizes of 13 × 13, 26 × 26, and 52 × 52. For the feature map with size of 13 × 13, the first detection result is obtained after convolution operation and entry into the detection layer. Then, up-sampling is conducted to obtain the feature map with size of 26 × 26 by fusing the map with the same-sized feature map in the previous network. The second detection result is obtained after convolution operation and entry into the detection layer again. Similarly, the feature map of 26 × 26 size is processed in the same manner to obtain the third detection result. Therefore, the multi-scale prediction of YOLOv3 has good adaptability for different scale detection objects, and feature expression ability is further enhanced.

**Figure 1:**
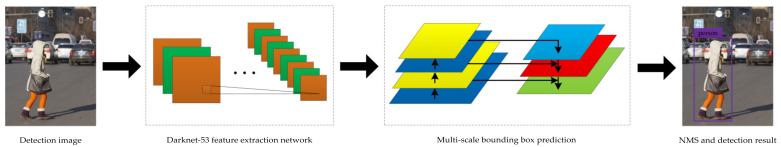
The schematic diagram of the detection process using YOLOv3.

Figure 1 shows the schematic diagram of the detection process using YOLOv3. The figure intuitively shows that, firstly, the detection image is preprocessed and sent to the Darknet-53 network for feature extraction. Then, three different scales of bounding box prediction are carried out in combination with the FPN network structure to detect whether a pedestrian object exists in the input image. Finally, the multiple confidence rates of bounding boxes are sorted and filtered, and NMS method is used to retain only the detection box with the maximum confidence, and ultimately output the detection result.

### 2.2. Limitations of YOLOv3 in Pedestrian Detection

YOLOv3 has fast detection speed while maintaining relatively high detection accuracy, and has become one of the object detection algorithms with good performance at present. However, in a variety of complex scenarios, some problems remain when the original YOLOv3 network model is applied to pedestrian detection, including inaccurate positioning of pedestrian objects, occlusion and small-scale pedestrians can easily cause missed detection. 

The causes of the above problems are analyzed and summarized as follows:

(1) In the actual road scenarios, the scale of pedestrian objects in the far and near fields of vision is different. At the beginning of training, the YOLOv3 network model needs to divide the detection image evenly. The size of the original grid cells is extremely large such that many small-scale pedestrian objects exist in the grid cells located in the far field of view, which can easily lead to missed detection.
(2) The original YOLOv3 network model used the k-means clustering algorithm to cluster pedestrian datasets at the initial stage of network training. In practical engineering applications, many human factors may cause invalid annotation data in the manual annotation dataset, resulting in poor matching between the prior box and pedestrian object, thereby exacerbating the complexity of the training network and extending the network training time.
(3) The size of the detection image captured by the vehicle-mounted camera is generally large, whereas the proportion of pedestrian object is small. In the actual driving environment, the shooting distance and angle of the same pedestrian object changes, and scaling and target rotation are likely to occur, resulting in a large change in pedestrian characteristics. As the information on small-scale pedestrians is easily lost in the deep-level feature map, the original YOLOv3 network model cannot fully identify the scale features of the pedestrian object, which leads to missed detection and object positioning deviation.
(4) When the intelligent vehicle passes through the pedestrian concentration areas such as intersections, crosswalks and hot spots, there may be dozens or even more pedestrian objects in a single detection image obtained, and pedestrians can easily block each other. If the pedestrian detection accuracy of smart car is not high enough at this time, it will undoubtedly pose a huge threat to the lives of many innocent pedestrians. The original YOLOv3 network model uses the NMS method to simply and roughly delete other detection boxes that have high overlap with the maximum confidence detection box, and also deletes some effective detection boxes, thereby reducing the detection accuracy of the algorithm in the object intensive scenario.

## 3. Improved YOLOv3 Network Model

### 3.1. Improved Grid Cell Size

In the original YOLOv3 network model, the detection image is evenly divided into grid cells with size of 7 × 7. The grid cell where the center of the pedestrian object is located is responsible for predicting the pedestrian object. Accurately increasing the division density of grid cells can help improve the detection accuracy of the network model and reduce the probability of missed detection of pedestrian objects. However, if the division density of grid cells is too high, it will play a counterproductive detection effect. Therefore, in order to determine an appropriate grid cell size, a set of control variable comparison experiments are conducted in this paper, based on INRIA person dataset for repeated training and testing. Under other conditions consistent, observe the pedestrian detection performance of YOLOv3 under different grid cell sizes, and the experimental results are shown in Table 2.

**Figure 2:**
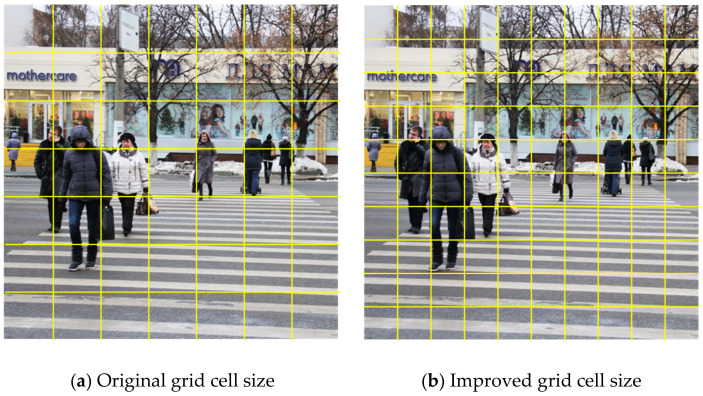
An example picture of improved grid cell size.

It can be seen from the above table that when the grid cell size is the original 7 × 7, the average processing time of each frame is the shortest, while the corresponding mean Average Precision (mAP) value is the lowest. When the grid cell size is 14 × 14, the corresponding mAP value is the highest, while the average processing time of each frame is the longest. Nevertheless, when the grid cell size is 10 × 10, the mAP value obtained is only slightly lower than the highest value, and the detection time is not extended too much, and it still has a faster running speed. Therefore, this study chooses 10 × 10, which is a relatively compromised experimental result, as the division size of the detection image, so that the pedestrian detection algorithm can still achieve detection efficiency while improving the detection accuracy. Figure 2 shows an example picture of improved grid cell size.

### 3.2. Improved k-Means Clustering Algorithm

The original YOLOv3 network model uses k-means clustering algorithm to perform the prior box unsupervised learning. This algorithm uses Euclidean distance as the evaluation index of object similarity in the clustering process, which is an iterative algorithm for automatic clustering. However, different sizes of the real box of the collected dataset may exist, and the error of the larger real box is larger than that of the smaller one in the iterative updating. Therefore, using the Euclidean distance as the evaluation index of object similarity in the unsupervised learning process of prior box is inaccurate [39]. As the ultimate goal of the prior box, unsupervised learning is to make the size of the detection box as close as possible to the size of the real box, this study selects *IOU* as the evaluation index to describe the distance between the real box and cluster center.

*IOU* is a commonly used metric, referring to the area ratio of the obtained detection and real boxes [40], which can be expressed as

(2) $$IOU=\frac{Gt\cap Dr}{Gt\cup Dr}$$

where *Gt* (ground truth) represents the real box of the object; *Dr* (detection result) represents the detection box of the object; Gt∩Dr represents the intersection of the real box and detection box; and Gt∪Dr represents the union of the real box and detection box.

The distance between the real box and cluster center can be expressed as follows:

(3) d(box,centroid)=1−IOU(box,centroid)

where *box* represents the real box and *centroid* represents the cluster center.

Equation (3) shows that the larger the *IOU* value between the real box and cluster center, the smaller is the distance between them. The k-means clustering algorithm based on *IOU* value can effectively reduce the error caused by the size of the real box, which is conducive to obtaining a more accurate cluster center value. 

Before using the k-means clustering algorithm to cluster the pedestrian dataset, the invalid annotation data in the dataset needs to be cleared. In this study, the width and height of the real box are taken as an important reference in the data filtering process. If points or lines exist in the dataset, then the corresponding width or height of the real box is 0, and the data are considered invalid. As the pedestrian objects are mostly thin and tall, the data are also considered invalid if the aspect ratio of the real box is greater than 3.

The basic steps of the improved k-means clustering algorithm are as follows:

(1) The invalid annotation data in the training dataset are eliminated.
  (1a) Coordinate data are written from the data file corresponding to the training dataset of the array.
  (1b) Read the array data in sequence. The projection coordinate of the vertex at the lower left corner of annotation box on the x axis is defined as $x_{\min}$. The projection coordinate on the y axis is defined as $y_{\min}$. The projection coordinate of the vertex at the upper right corner of the annotation box on the x axis is defined as $x_{\max}$, and the projection coordinate on the y axis as $y_{\max}$.
  (1c) The difference between $x_{\max}$ and $x_{\min}$ is calculated and recorded as $x_{d}$. The difference between $y_{\max}$ and $y_{\min}$ is recorded and calculated as $y_{d}$. If $x_{d}=0$ or $y_{d}=0$, then the annotation data corresponding to $x_{d}$ and $y_{d}$ is invalid; otherwise, it is valid.
  (1d) The quotient of $x_{d}$ and $y_{d}$ is calculated and recorded as Q. If Q>3, then the annotation data corresponding to $x_{d}$ and $y_{d}$ is invalid; otherwise, it is valid.
  (1e) All valid annotation data in the training dataset are obtained.
(2) Effective annotation data are clustered.
  (2a) The *k* clusters are artificially selected and *k* initial clustering centers are randomly selected.
  (2b) The *IOU* values of all valid annotation data and clustering centers are calculated.
  (2c) The data points with larger *IOU* value are automatically divided into the cluster where the cluster center is located.
  (2d) The center of all data points in each cluster is selected as the new clustering center.
  (2e) Steps (2b)–(2d) are repeated until the cluster center no longer moves.
(3) The final clustering result is used as the prior box obtained by unsupervised learning of the YOLOv3 network model.

The improved k-means clustering algorithm can almost completely eliminate the effect of invalid annotation data on the clustering center, greatly improving the matching degree between the prior box and pedestrian object. This condition is not only beneficial to reduce the complexity of the training network and shorten the network training time but also helps to improve the detection accuracy of the YOLOv3 network model.

### 3.3. Improved Multi-Scale Bounding Box Prediction Based on Receptive Field

The original YOLOv3 network model uses the feature extraction network with deep convolution. However, during the network training process, as the number of network layers gradually deepens, the relevant information on small-scale pedestrian objects is increasingly lost [41]. Therefore, expanding the receptive field of deep convolutional layers as much as possible is necessary to improve the feature recognition level of the network model for pedestrian objects of different scales. Receptive field refers to the size of the area where the pixels on the feature map output by each layer in the CNN are mapped on the original input image. As the convolutional kernels of 1 × 1 and 3 × 3 are widely used in the down-sampling process of YOLOv3, the receptive field increases gradually as the network depth increases. It is a relative concept, and the calculation formula can be expressed as follows:

(4) $$RF_{i}=s_{i}\times (RF_{i-1}-1)+k_{i}$$

where *RF* represents the size of receptive field; *s* is the convolution step size; *k* is the size of convolution kernel; and *i*, *i*-1 are the number of convolutional layers. 

In the original multi-scale bounding box prediction, the last layer feature maps of size 13 × 13, 26 × 26, and 52 × 52 are fused with the same-size feature map obtained by up-sampling, and the detection results of this size feature layer are obtained. According to Equation (4), the receptive field size of the last feature layer of relevant size in the original YOLOv3 is reported in Table 3.

To fully utilize the large amount of semantic information on high-level features and detailed information of low-level features, this study improves the multi-scale bounding box prediction. On the basis of the original three-scale detection module, the feature map with size of 52 × 52 is up-sampled to obtain the feature map with size of 104 × 104. The new feature map is obtained by fusing with the feature map of the same size in the shallow network, and the fourth detection result is obtained after convolution operation and entry into the detection layer. As the number of convolutional layers with 104 × 104 size in the shallow network is small, based on the original Darknet-53 network, six convolutional layers with 104 × 104 size are added to the shallow network to achieve an improved detection effect. Table 4 presents the receptive field size of the last feature layer of relevant size in the improved YOLOv3.

A comparison of Table 3 and Table 4 show intuitively that in the improved YOLOv3 network model, the receptive field size of the last feature layer of four sizes have been significantly increased. In particular, for the feature layer with 104 × 104 size, the corresponding receptive field size has increased from 29 × 29 to 77 × 77. Improving the multi-scale bounding box prediction based on receptive field is beneficial to enhance the network’s ability to pay attention to global information, effectively identify the scale features of pedestrian objects, improve the detection accuracy of the network model for small-scale pedestrian objects, and greatly reduce the occurrence of missing detection.

### 3.4. Soft-NMS Algorithm

The objective of the traditional NMS algorithm is to search for local maxima, suppress non-maximum elements, and complete the main operation based on the obtained confidence of the detection boxes and overlap between the detection boxes. Two main problems exist in the traditional NMS algorithm. First, when the two detection boxes are relatively close, the effective detection box with a slightly lower confidence is deleted only because of its large overlapping area. Second, the overlap threshold needs to be an artificial setting; if the setting is extremely large, then it will cause false detection, and if the setting is extremely small, then it will cause missed detection. Therefore, the NMS algorithm completely relies on the confidence of the detection box and simply deletes other detection boxes that are larger than the overlap threshold, which cannot achieve the ideal pedestrian detection effect. 

Considering the shortcomings of the traditional NMS algorithm, this study uses the Soft-NMS algorithm as the detection box selection scheme. This algorithm does not directly delete all detection boxes whose *IOU* is larger than the threshold, but reduces their confidence. The larger the *IOU* between the detection box to be processed and the detection box with the current maximum confidence, the faster is the decrease in the confidence of the detection box to be processed. According to the actual situation, the Soft-NMS algorithm selects one of two penalty functions, linear and Gaussian, to attenuate the confidence of the detection box.

The linear penalty function is defined as follows:

(5) $$s_{i}=\left\{\begin{array}{cc} s_{i}, & iou(M,b_{i})<N_{t}\\ s_{i}(1-iou(M,b_{i})), & iou(M,b_{i})\geq N_{t} \end{array}\right.$$

where $s_{i}$ is the confidence of the detection box to be processed, *M* represents the detection box with the current maximum confidence, $b_{i}$ represents the detection box to be processed, and $N_{t}$ is the overlap threshold.

The Gaussian penalty function is defined as follows:

(6) $$s_{i}=\begin{array}{cc} s_{i}e^{-\frac{iou{\left(M,bi\right)}^{2}}{\sigma }}, & \forall b_{i}\notin D \end{array}$$

The Soft-NMS algorithm is a more general non-maximum suppression algorithm that does not require retraining of the network model, is easy to implement, and can be effectively applied to improved pedestrian detection algorithm. Compared with the traditional NMS algorithm, the Soft-NMS algorithm improves the accuracy of the pedestrian object positioning, has good adaptability in the object intensive scenario, which is helpful to further improve the detection performance of the network model.

## 4. Pedestrian Detection Experiment and Discussion

### 4.1. Experimental Environment

Software environment: Windows 10 64-bit operating system, CUDA 9.1, cuDNN 7.1, Darknet framework, and Python 3.7.0.

Hardware environment: Intel (R) Core (TM) i7-7700 CPU@3.60GHz processor, 32 GB memory, and NVIDIA GeForce GTX 1080Ti GPU, 11 GB.

### 4.2. Pedestrian Detection Experiment

#### 4.2.1. Pedestrian Dataset

**Figure 3:**
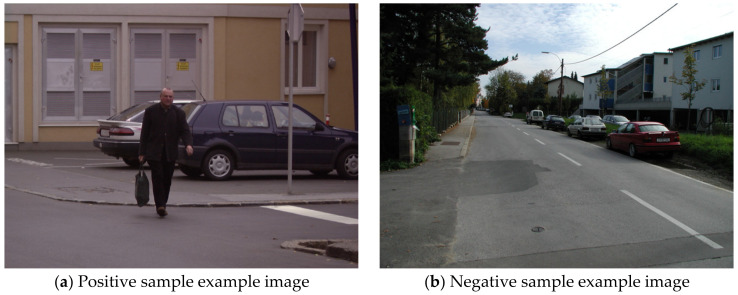
The sample example image of INRIA person dataset.

In this study, INRIA person dataset is used for pedestrian detection experiment. This dataset was first proposed by Dalal, mainly from GRAZ-01, personal photos, and Google, and it has become a static pedestrian dataset widely used by experts and scholars in computer vision and assisted driving [42,43]. The INRIA person dataset is divided into two parts: training set and testing set, and each part consists of positive samples including pedestrians and negative samples excluding pedestrians. Among them, the training set includes 614 positive sample images (containing 2416 pedestrian objects) and 1218 negative sample images, and the test set includes 288 positive sample images (containing 1126 pedestrian objects) and 453 negative sample images. Figure 3 presents the sample example image of INRIA person dataset, in which (a) is a positive sample example image and (b) is a negative sample example image.

The internal images of INRIA person dataset vary in size and have high definition, and the minimum and maximum images are 640 × 480 and 1280 × 960 pixels, respectively. The pedestrians in the image are mostly standing, and the height is not less than 100 pixels, which meets the actual detection needs. Pedestrian objects in the dataset are in various complex scenarios, including streets, mountains, and beaches. Pedestrians in the image have different scales and postures, and may block each other. Therefore, INRIA person dataset has a high degree of authenticity, which can reflect the pedestrian characteristics in the actual road scenarios, and is conducive to fully testing the pedestrian detection performance of the improved YOLOv3.

Considering the small number of sample images in the INRIA person dataset, there may be over-fitting phenomenon in the training process, thereby affecting the training effect of the network model. To solve this problem effectively, this study applies the method of data enhancement to generate an artificial dataset. By translating, rotating, mirroring, cropping, adding noise, and adjusting brightness, the INRIA training set can be expanded to 20,000 detection images.

#### 4.2.2. Network Training and Evaluation Indicators

**Figure 4:**
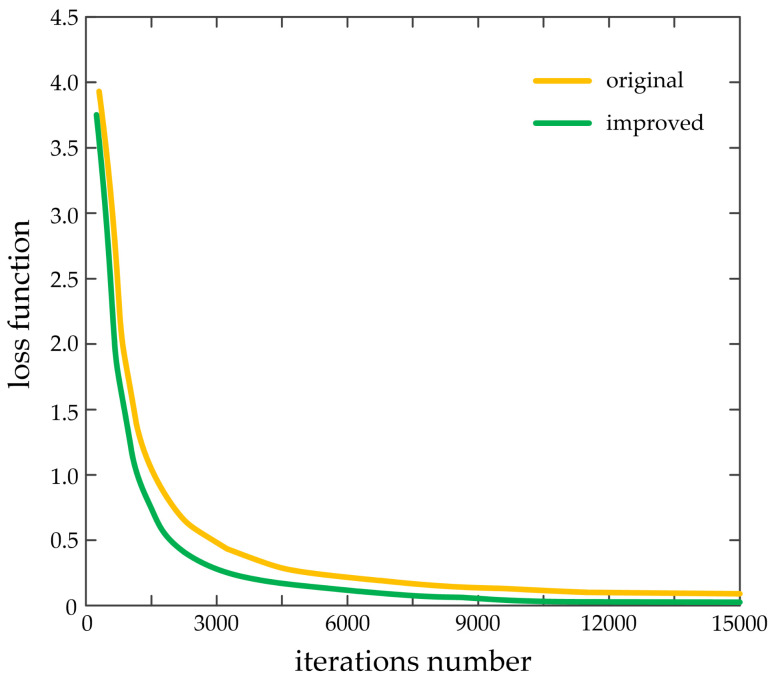
The change curves of loss function about original and improved YOLOv3.

The network training is based on the improved YOLOv3 network model. The initial learning rate is set to 0.001, and the learning rate is adjusted based on the polynomial decay strategy during the training process. The weight decay coefficient is set to 0.001 and the momentum coefficient is set to 0.1. The batch normalization is used to prevent over-fitting, and the size of the input image is randomly changed every 10 iterations. The Leaky ReLU function is still used as the activation function, and the mean-square error loss function is used as loss function. Figure 4 shows the change curves of loss function about original and improved YOLOv3.

According to the preceding figure, compared with the original YOLOv3, the loss function of the improved YOLOv3 converges more quickly, and gradually decreases to 0. The loss function value of improved YOLOv3 is generally lower than that of the original YOLOv3. When the iterations number is 2200, the corresponding loss function difference reaches the maximum. When the network iterates to the maximum iterations number of 15,000, the corresponding loss function difference reaches the minimum. In general, the improved YOLOv3 network model improves the convergence speed of the training network, and effectively enhances the overall stability of the training network.

As the detected image has positive and negative samples, four types of model predictions are available: the real is a positive sample and the prediction is a positive sample (True Positive); the real is a positive sample and the prediction is a negative sample (False Negative); the real is a negative sample and the prediction is a negative sample (True Negative); and the real is a negative sample and the prediction is a positive sample (False Positive). Therefore, Precision, Recall, Average Precision (AP), and mean Average Precision (mAP) can be calculated and used as the evaluation indicators to measure the detection performance of the model.

Precision refers to the ratio between the number of correctly detected objects and the number of all detected objects, expressed by the following formula:

(7) $$P=\frac{TP}{TP+FP}$$

Recall refers to the ratio between the number of correctly detected objects and the number of all labeled objects, expressed by the following formula:

(8) $$R=\frac{TP}{TP+FN}$$

AP refers to the average value of the correct detection for a certain category of objects, which can be obtained by calculating the integral function using Precision and Recall, and expressed by the following formula:

(9) $$AP=\int_{0}^{1}P(R)dR$$

mAP refers to the average value of the average precision of all categories of objects, used to evaluate the overall detection accuracy of the model, and can be expressed by the following formula:

(10) $$mAP=\frac{\sum AP}{N}$$

where *N* is the number of objects in all categories.

#### 4.2.3. Experimental Test Results and Analysis

As the overlap threshold is artificially set, different overlap threshold settings may produce different Precision and Recall for the same dataset. Therefore, determining an optimal overlap threshold is necessary. After multiple tests, the results under different overlap thresholds are shown in Table 5, where F1-score represents the comprehensive score rate based on the Precision and Recall.

In this study, 15 overlap thresholds are selected for testing, starting from 0.05, increasing 0.05 each time until 0.75 is reached. The table shows that as the overlap threshold gradually increases, the Precision gradually increases and the Recall gradually decreases, thereby showing an inverse relationship between the two. When the overlap threshold is 0.20, the Precision is 93.74% and the Recall is 88.14%. At this time, the F1-score is 90.85%, and the comprehensive detection effect is the best. Therefore, the overlap threshold is set to 0.20.

To test and verify the detection performance of the improved YOLOv3 network model, the pedestrian detection test is carried out by using the testing set. The test results of some typical detection images are shown in the figure below, where (a) indicates the pedestrian detection test results based on the original YOLOv3 network model, and (b) shows the pedestrian detection test results based on the improved YOLOv3 network model.

**Figure 5:**
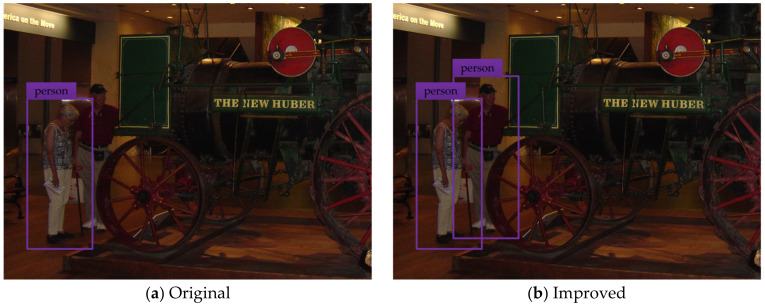
Pedestrian detection test results in the dim scenario.

**Figure 6:**
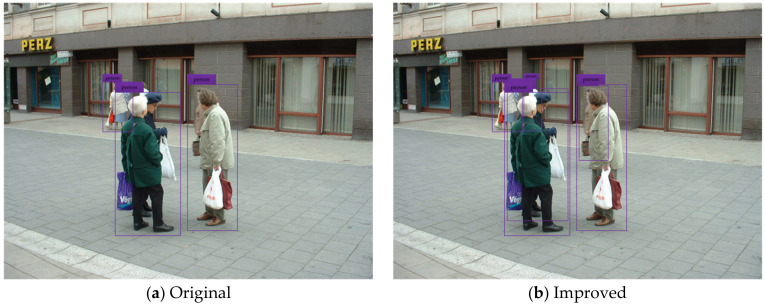
Pedestrian detection test results under occlusion.

**Figure 7:**
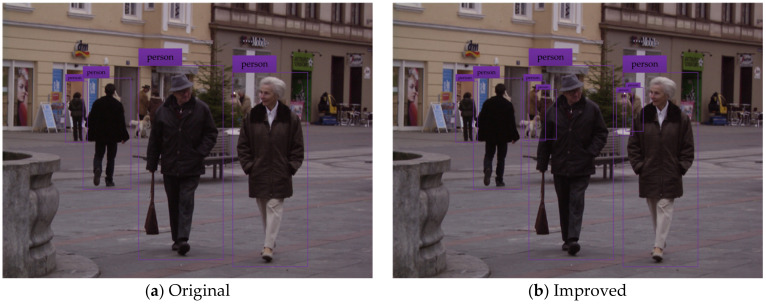
Pedestrian detection test results in the multi-scale scenario.

**Figure 8:**
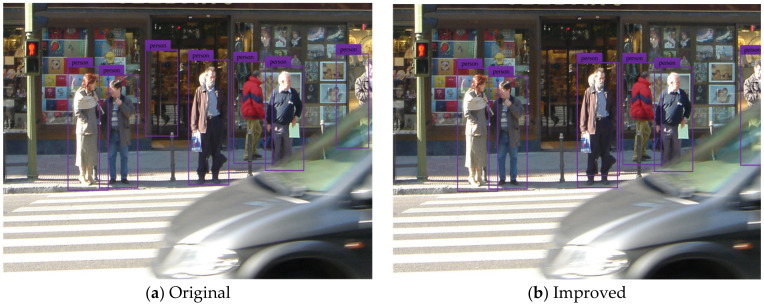
Pedestrian detection test results under complex background.

**Figure 9:**
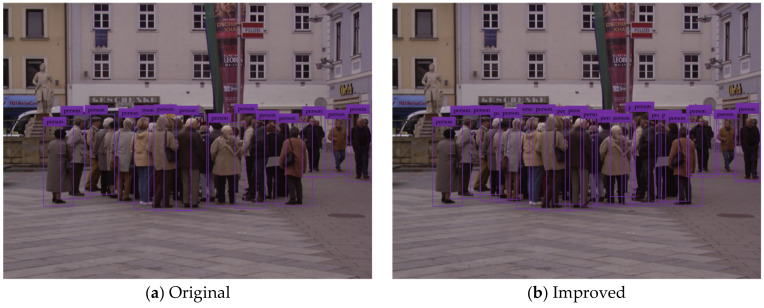
Pedestrian detection test results in the object intensive scenario.

Figure 5, Figure 6, Figure 7, Figure 8 and Figure 9 show the pedestrian detection test results in various complex scenarios. Figure 5 shows that when the original YOLOv3 network model is used for pedestrian detection, the pedestrian objects are in the dim scenario, and the pedestrian on the right is partially blocked by the train model, resulting in the missed detection of this person, whereas the improved network model realizes the effective detection of all pedestrian objects. Figure 6 indicates that when some pedestrian objects are blocked by other pedestrians, the original network model causes missed detection for pedestrian objects with a greater degree of occlusion, while the improved network model achieves good detection for the pedestrian objects with different degrees of occlusion. In Figure 7, as the small-scale pedestrian objects contain fewer features and are relatively fuzzy, they are not easily captured by the network. The original network model caused missed detection of four pedestrian objects in the detection image, while the improved network model accurately detects all pedestrian objects in the picture. Figure 8 shows that as pedestrian objects are affected by background factors such as oil painting, glass, and commodities, the original network model causes a false detection of the detection image, and the positioning of the rightmost pedestrian object is inaccurate. Nevertheless, the improved network model can effectively avoid false detection and minimize the positioning deviation of pedestrian objects. Figure 9 shows that many pedestrians are in the object intensive scenario because the pedestrian objects are mostly back views and mutual occlusion occurs between them. Thus, the network cannot completely extract the object features. The original network model causes a large number of missed detections on the detection image, and in some cases, the object positioning is inaccurate. The improved network model greatly improves the aforementioned situation, and realizes the effective detection and accurate positioning of all pedestrian objects.

Pedestrian detection test results show that the improved YOLOv3 network model has excellent detection performance. In a variety of complex scenarios, the proposed pedestrian detection algorithm has good adaptability to different environment and background conditions. Compared with the original YOLOv3 network model, the improved model is enhanced in terms of detection accuracy and network stability, showing superior robustness and anti-interference ability, which is conducive to achieving the accurate detection of pedestrian objects ahead of smart cars.

### 4.3. Object Detection Based on PASCAL VOC 2012 Dataset

In addition to applying the INRIA person dataset for pedestrian detection experiments, this study also uses the PASCAL VOC 2012 dataset for performance testing. This dataset is the most representative general object detection dataset so far, and is used to identify specific objects from real-world images. The PASCAL VOC 2012 dataset is an upgraded version of the PASCAL VOC 2007 dataset, which is mainly used to achieve the three tasks of object classification, detection, and segmentation [44,45]. With regard to the detection task, the trainval/test part of the dataset covers all relevant images from 2008 to 2011. The trainval part contains 11,540 detection images, representing a total of 27,450 objects. In the detection image, 4 major categories and 20 minor categories of objects exist. In addition to testing all the pedestrian objects in the dataset, this study also tests other objects such as car, bus, bicycle and motorbike that often appear in daily road traffic, to further test the comprehensive detection performance of the proposed algorithm. Table 6 shows the object detection test results of various types of objects in the actual road scenarios.

The table shows that the detection accuracy of the original YOLOv3 network model for pedestrian objects is 79.20%, whereas that of the improved network model for pedestrian objects is 90.60%, and the detection performance has been further improved. In the improved mAP, the detection accuracy of the bus is the highest, reaching 94.10%, and that of the motorbike is the lowest, reaching 86.30%, and the detection accuracy of different types of objects has been improved to varying degrees. Overall, the average detection accuracy of the original YOLOv3 network model for all objects is 84.00%, whereas the improved network model for all objects is 91.14%. The data show that the proposed algorithm based on PASCAL VOC 2012 dataset has superior generalization ability and comprehensive object-detection performance, which can be widely used in the accurate detection of a variety of objects.

### 4.4. Performance Comparison of Detection Algorithms

To verify the performance of the pedestrian detection algorithm, the proposed algorithm is compared with algorithms used in other studies. Table 7 lists the comparison of statistics in algorithm performance based on the INRIA person dataset.

As shown in the table, the proposed algorithm and other studies conducted relevant pedestrian detection experiments based on the INRIA person dataset. The evaluation indicators for performance comparison of the detection algorithm mainly include mAP value and the average processing time per frame. In reference [46], a pedestrian detection algorithm combining aggregate channel features and CNN was adopted. When only the ACF detector was used in comparison with other algorithms, although the average processing time was relatively short, the mAP value was the lowest. Then, when the ACF detector cascaded the CNN architecture, although the mAP value had increased, the average processing time was extremely long and could not be effectively applied to real-time pedestrian detection for intelligent vehicles. In reference [47], a pedestrian detection method combining the histogram of the oriented gradient and discrete wavelet transform was utilized. This method used the magnitude of motion to set the region of interest, effectively reducing the computational complexity. However, the method did not obtain satisfactory detection accuracy while achieving a very high detection speed, and further improvement was needed. In addition to using the improved YOLOv3 network model for pedestrian detection, this study also uses the original YOLOv3 network model for testing based on the INRIA person dataset to reflect the improvement of detection performance. The average processing time corresponding to the original YOLOv3 network model is comparatively short, but the mAP value is still relatively low, which is prone to missed or false detection when applied to the actual road scenarios. Compared with the aforementioned studies and algorithms, the proposed algorithm has the best comprehensive detection performance, the mAP value reaches 90.42%, and the average processing time of each frame is 9.6 ms. The improved YOLOv3 network model exhibits accuracy and real-time performance together in pedestrian detection, which is beneficial to fully meet the accuracy and real-time target requirements of pedestrian detection for smart cars in the actual road scenarios, thereby helping to further protect the road safety of pedestrians and improve the technological level of intelligent vehicle driving assistance.

## 5. Conclusions

In this study, a pedestrian detection algorithm for intelligent vehicle in complex scenarios is proposed. First, the basic principle of YOLOv3 is elaborated and analyzed to determine its limitations in pedestrian detection. Then, on the basis of the original YOLOv3 network model, many improvements are made, including modifying grid cell size, adopting improved k-means clustering algorithm, improving multi-scale bounding box prediction based on receptive field, and using Soft-NMS algorithm. Finally, based on INRIA person and PASCAL VOC 2012 datasets, pedestrian detection experiments are conducted to test the performance of the algorithm in various complex scenarios. The experimental results show that the mAP value reaches 90.42%, and the average processing time of each frame is 9.6 ms. Compared with other detection algorithms, the proposed algorithm exhibits accuracy and real-time performance together, good robustness and anti-interference ability in complex scenarios, strong generalization ability, high network stability, and detection accuracy and detection speed have been markedly improved.

From the perspective of pedestrian detection accuracy and operating efficiency, the proposed algorithm has large advantages, which meet the accuracy and real-time target requirements of pedestrian detection for smart cars in the actual road scenarios. These advantages are also important in protecting the road safety of pedestrians and ensuring the steady development of the technological level of intelligent vehicle driving assistance. In the future, the pedestrian detection algorithm under severe working conditions and algorithm hardware transplantation can be conducted in-depth research, so as to improve the overall performance and practical application value of the algorithm.

## Figures and Tables

**Table 1:** The basic parameters of the Darknet-53 network framework.

Processing Mode	Residual Block Number (n)	Step Size	Convolution Kernel Number	Output Scale
Conv_BN		1	32	416 × 416
Conv_BN		2	64	208 × 208
Res_Conv_n	1	1	64	208 × 208
Conv_BN		2	128	104 × 104
Res_Conv_n	2	1	128	104 × 104
Conv_BN		2	256	52 × 52
Res_Conv_n	8	1	256	52 × 52
Conv_BN		2	512	26 × 26
Res_Conv_n	8	1	512	26 × 26
Conv_BN		2	1024	13 × 13
Res_Conv_n	4	1	1024	13 × 13

**Table 2:** The pedestrian detection performance of YOLOv3 under different grid cell sizes.

Sequence Number	Grid Cell Size	mAP (%)	Average Processing Time (ms)/Frame
1	7 × 7	83.54	13.5
2	10 × 10	85.22	13.8
3	14 × 14	85.25	15.1

**Table 3:** The receptive field size of the last feature layer of relevant size in the original YOLOv3.

Convolutional Layer	Receptive Field Size	Feature Layer Size	Description
11th_layer	29 × 29	104 × 104	-
36th_layer	165 × 165	52 × 52	Output Layer
61th_layer	437 × 437	26 × 26	Output Layer
82th_layer	917 × 917	13 × 13	Output Layer

**Table 4:** The receptive field size of the last feature layer of relevant size in the improved YOLOv3.

Convolutional Layer	Receptive Field Size	Feature Layer Size	Description
29th_layer	77 × 77	104 × 104	Output Layer
54th_layer	213 × 213	52 × 52	Output Layer
79th_layer	485 × 485	26 × 26	Output Layer
100th_layer	965 × 965	13 × 13	Output Layer

**Table 5:** The multiple test results under different overlap thresholds.

Sequence Number	Overlap Threshold	Precision (%)	Recall (%)	F1-Score (%)
1	0.05	83.58	93.06	88.05
2	0.10	89.14	91.38	90.23
3	0.15	91.98	89.67	90.80
4	0.20	93.74	88.14	90.85
5	0.25	95.09	86.60	90.64
6	0.30	96.01	85.09	90.21
7	0.35	96.76	83.58	89.68
8	0.40	97.32	81.96	88.97
9	0.45	97.70	80.24	88.12
10	0.50	98.27	78.42	87.13
11	0.55	98.38	76.27	85.96
12	0.60	98.65	73.96	84.54
13	0.65	98.93	71.51	82.98
14	0.70	99.12	68.45	80.99
15	0.75	99.26	64.93	78.52

**Table 6:** The object detection test results of various types of objects in the actual road scenarios.

Sequence Number	Object Type	Original mAP (%)	Improved mAP (%)
1	Person	79.20	90.60
2	Car	85.90	92.80
3	Bus	86.70	94.10
4	Bicycle	84.00	91.90
5	Motorbike	84.20	86.30
Total	-	84.00	91.14

**Table 7:** The comparison of statistics in algorithm performance based on the INRIA person dataset.

Sequence Number	Method	mAP (%)	Average Processing Time (ms)/Frame	System Environment
1	ACF [46]	83.17	65.9	Intel Core i7-4710 HQ@2.50 GHz, 12 GB RAM
2	ACF + CNN [46]	84.87	295.9	Intel Core i7-4710 HQ@2.50 GHz, 12 GB RAM
3	HOG + DWT [47]	85.12	1.5	Machine of 3.4 GHz CPU
4	Original YOLOv3	83.54	13.5	Intel(R) Core(TM) i7-7700 CPU@3.60GHz
Ours	Improved YOLOv3	90.42	9.6	Intel(R) Core(TM) i7-7700 CPU@3.60GHz
